# Comparison of Emergence Agitation Between Remimazolam- and Propofol-Based Anesthetic Induction in Patients Undergoing Transurethral Endoscopic Urologic Surgery: A Propensity Score-Matched Retrospective Study

**DOI:** 10.7150/ijms.137664

**Published:** 2026-07-22

**Authors:** Ji-Yoon Jung, Hwang-Ju You, Woojin Kwon, Junho Kim, Tae-Yun Sung

**Affiliations:** Department of Anesthesiology and Pain Medicine, Konyang University Hospital, Konyang University Myunggok Medical Research Institute, Konyang University College of Medicine, 158 Gwangeodong-ro, Seo-gu, Daejeon 35365, Republic of Korea.

**Keywords:** emergence delirium, remimazolam, propofol, urologic surgical procedures, anesthesia, general

## Abstract

**Background:**

Emergence agitation is a clinically relevant phenomenon during recovery from general anesthesia. This study aimed to compare the effects of remimazolam and propofol as anesthetic induction agents on emergence agitation.

**Methods:**

This retrospective propensity score-matched study included adult patients undergoing elective transurethral endoscopic urologic surgery under general anesthesia. Patients were divided into remimazolam- or propofol-based induction groups. The primary outcome was the incidence of emergence agitation, defined as a Ricker Sedation-Agitation Scale (RSAS) score ≥ 5 during emergence. Secondary outcomes included the severity of emergence agitation, extubation time, postoperative pain scores, use of analgesics or antiemetics in the post-anesthesia care unit (PACU) and risk factor identification.

**Results:**

A total of 239 patients were analyzed before matching, and 99 matched pairs were generated. After matching, the incidence of emergence agitation was significantly lower in the remimazolam group compared with the propofol group (13.1% vs. 36.4%; P = 0.001). Remimazolam-based induction was independently associated with a reduced risk of emergence agitation (odds ratio 0.23, 95% confidence interval 0.09-0.56; P < 0.001). The distribution of RSAS scores demonstrated reduced severity of agitation in the remimazolam group. Extubation time, postoperative pain scores, and PACU medication did not differ between groups. Younger age and male sex were identified as independent risk factors.

**Conclusions:**

Remimazolam-based anesthetic induction reduced incidence and severity of emergence agitation compared with propofol, without delaying extubation or impairing early postoperative recovery. Remimazolam may represent a favorable induction agent for adult patients at risk for emergence agitation.

## Introduction

Emergence agitation is a clinically relevant phenomenon occurring during recovery from general anesthesia and is characterized by restlessness, disorientation, and non-purposeful movement. Although often transient, emergence agitation may result in self-injury, accidental removal of catheters or airway devices, hemodynamic instability, and increased workload in the post-anesthesia care unit (PACU) 1. While emergence agitation has been extensively studied in pediatric populations, it is increasingly recognized as a significant issue in adult surgical patients, particularly in procedures associated with postoperative discomfort or urinary catheterization, such as urologic surgery 2, 3.

Propofol is widely used as a standard agent for anesthetic induction because of its rapid onset and favorable recovery profile. Nevertheless, emergence agitation remains a clinically relevant problem during recovery from general anesthesia, even when propofol is used for induction, and is influenced by patient-related factors, surgical characteristics, anesthetic management, and perioperative pain 1, 4. In adults, male sex and younger age have been consistently reported as risk factors for emergence agitation, whereas strategies to reliably prevent emergence agitation remain limited 2-5. Therefore, optimizing anesthetic techniques to reduce emergence agitation without prolonging recovery time is of substantial clinical interest.

Remimazolam is an ultra-short-acting benzodiazepine with organ-independent metabolism by tissue esterases, offering rapid onset, predictable recovery, and hemodynamic stability 6. Several randomized controlled trials and pooled analyses have demonstrated that remimazolam is a safe and effective agent for the induction and maintenance of general anesthesia, with more favorable hemodynamic profiles 7, 8. In addition, randomized studies in procedural sedation have consistently shown greater cardiovascular stability with remimazolam, supporting its potential advantages over propofol across a range of clinical settings 9, 10.

Given the pharmacological properties of benzodiazepines, which include anxiolytic and sedative effects, remimazolam may theoretically attenuate emergence agitation. Although the clinical and pharmacologic profile of remimazolam has been described in procedural sedation studies and pharmacokinetic/pharmacodynamic analyses 11,12, evidence directly evaluating its effect on emergence agitation remains limited. A previous randomized study evaluated remimazolam in relation to emergence agitation during general anesthesia; however, evidence specifically addressing the effect of remimazolam when used only as an induction agent remains scarce 13.

We hypothesized that remimazolam-based induction would be associated with a lower incidence and severity of emergence agitation without delaying recovery. Accordingly, this retrospective study aimed primarily to compare the incidence of emergence agitation between remimazolam- and propofol-based anesthetic induction in adult patients undergoing elective transurethral endoscopic urologic surgery. Secondary objectives were to compare the severity of emergence agitation and extubation time between the two induction agents and to identify risk factors for emergence agitation.

## Methods

### Study design and population

This retrospective study was approved by the Institutional Review Board of Konyang University Hospital, Daejeon, Korea (approval number: KYUH 2025-12-042). This study was reported in accordance with the STROBE statement. Written informed consent was waived due to the retrospective nature of the study. The medical records of patients aged ≥ 19 who underwent elective transurethral endoscopic urologic surgery under general anesthesia in our hospital between June 2024 and February 2025 were reviewed retrospectively. A patient was excluded if any of the following criteria were met: (1) American Society of Anesthesiologists physical status classification ≥ IV; (2) body mass index of > 35 kg/m^2^; (3) use of supraglottic airway device; (4) combined operation; (5) cognitive or neuropsychological disorder; (6) total intravenous anesthesia; (7) missing essential preoperative laboratory data required for baseline assessment and data completeness, including hemoglobin, platelet count, serum creatinine, electrolytes, and liver function tests. Patients were divided into remimazolam or propofol groups according to the anesthetic induction agent used.

### Anesthetic protocol

All patients were fasted for at least 8 hours and arrived in the operating room without premedication. All patients received the same anesthetic management according to the standard institutional protocol, except for the anesthetic induction agent, remimazolam or propofol.

After application of routine monitoring, remimazolam was administered as a continuous infusion at 6 mg/kg/h until loss of consciousness was achieved in the remimazolam group, whereas propofol was administered as a bolus at a dose of 1.5-2 mg/kg in the propofol group. The remimazolam infusion was discontinued immediately after loss of consciousness, and no additional remimazolam was administered during maintenance or emergence. In both groups, fentanyl (1-2 μg/kg) was injected at the start of remimazolam infusion or immediately after propofol bolus administration. Rocuronium 0.6 mg/kg was administered after loss of consciousness was confirmed. Endotracheal intubation was performed at neuromuscular train-of-four (TOF) count 0. Anesthesia was maintained with sevoflurane in a 50% oxygen and 50% nitrous oxide mixture. Sevoflurane was adjusted to keep the Patient State Index (PSI; SedLine®; Masimo Corp., Irvine, CA, USA) of 25-50 during surgery. All patients underwent surgery in the lithotomy position. At the end of the operation, urinary catheterization was performed in all patients. Subsequently, patients were placed in the supine position, and the administration of inhaled anesthetics was discontinued. Flumazenil was not used in any patient. Patients received sugammadex for reversal of neuromuscular blockade and were transferred to the postanesthesia care unit (PACU) after extubation.

### Primary and secondary outcomes

The primary outcome of this study was the incidence of emergence agitation, defined as an RSAS score ≥ 5 during the emergence period. Secondary outcomes included the severity of emergence agitation, assessed by the highest Ricker Sedation-Agitation Scale (RSAS) score recorded during emergence; extubation time, defined as the interval from discontinuation of anesthetics to tracheal extubation; postoperative pain intensity assessed using the numeric rating scale (NRS) in the PACU; and the use of analgesics or antiemetic drugs in the PACU.

### Data collection and outcome variables

In our hospital, since January 2017, the attending anesthetist (nurse) routinely evaluates emergence agitation during emergence (i.e., from the discontinuation of anesthetics to 5 minutes after extubation) and records it on the electronic medical record. Emergence agitation was assessed using the Ricker Sedation-Agitation Scale (RSAS, 7 points; 1 = unarousable, 2 = very sedated, 3 = sedated, 4 = calm and cooperative, 5 = agitated but responding calmly to verbal instructions, 6 = very agitated requiring restraint, 7 = pulling at the tracheal tube, trying to remove catheter or striking the staff) 14. During emergence, the highest RSAS score was recorded and RSAS ≥ 5 was considered as an occurrence of emergence agitation.

The following data were collected and analyzed: age, sex, weight, height, body mass index (BMI), American Society of Anesthesiologists (ASA) physical status, duration of anesthesia, extubation time, administered crystalloid, estimated blood loss, use of postoperative patient-controlled analgesia (PCA), occurrence of emergence agitation, RSAS score, NRS pain score in PACU, analgesics or antiemetic drugs administration in PACU, vital sign at four time points (baseline, after anesthetics off, at extubation, 3 minutes after extubation).

### Statistical analysis

Demographic data were compared between groups before and after propensity score matching (PSM). PSM was performed to minimize baseline imbalances between patients receiving remimazolam and propofol for anesthetic induction. Patients were matched using 1:1 nearest-neighbor matching without replacement, with a caliper width of 0.2 of the standard deviation of the logit of the propensity score. Propensity scores were estimated using a multivariable logistic regression model incorporating possible risk factors of emergence agitation (i.e., age, sex, body mass index (BMI), ASA physical status, and duration of anesthesia) as covariates 5, 15. Covariate balance before and after matching was assessed using standardized mean differences (SMDs), with an absolute SMD < 0.1 considered indicative of adequate balance.

Continuous variables were summarized as mean ± standard deviation or median [interquartile range], depending on data distribution. Normality was assessed using the Kolmogorov-Smirnov test. Before matching, continuous variables were compared using Student's t-test or the Mann-Whitney U test, as appropriate. Categorical variables were presented as counts and percentages and compared using the chi-square test or Fisher's exact test, depending on expected cell counts.

After propensity score matching, all between-group comparisons were performed with consideration of the matched-pair structure. For continuous variables, paired t-tests were used for normally distributed data and Wilcoxon signed-rank tests were used for non-normally distributed data. For binary categorical variables, McNemar's test was used. The primary outcome, the incidence of emergence agitation, was additionally analyzed using conditional logistic regression to estimate the odds ratio and 95% confidence interval for remimazolam-based induction compared with propofol-based induction. For ordinal categorical variables, including the distribution of Ricker Sedation-Agitation Scale scores, the Wilcoxon signed-rank test was used to compare the paired distributions between groups. The distribution of Ricker Sedation-Agitation Scale scores after matching was illustrated using stacked bar plots.

To identify independent predictors of emergence agitation in the unmatched cohort, univariate and multivariable logistic regression analyses were performed. Variables with P < 0.1 in the univariate analysis were included in the multivariable analysis. A backward stepwise selection procedure was applied to derive the final model.

All statistical analyses were performed using R software version 4.6.0 (R Foundation for Statistical Computing, Vienna, Austria). Propensity score matching was performed using the MatchIt package. Conditional logistic regression was performed using the survival package. A two-sided P value < 0.05 was considered statistically significant.

## Results

**Figure 1:**
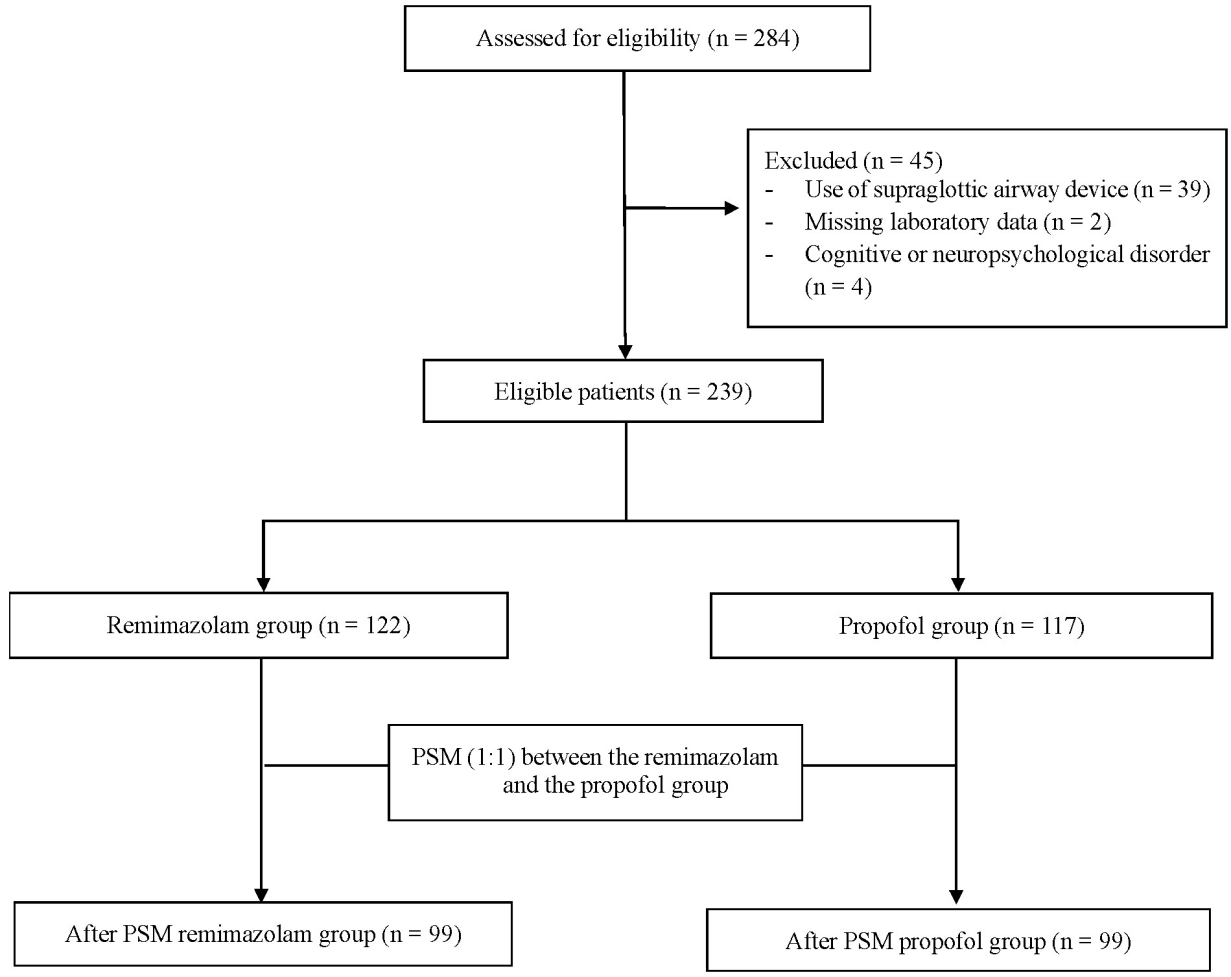
Flowchart of patient enrollment and propensity score matching.

From June 2024 to February 2025, a total of 284 patients aged 19 years or older who underwent elective transurethral endoscopic urologic surgery under general anesthesia at Konyang University Hospital were assessed. Among them, 45 were excluded, 239 patients were included in the analysis, comprising 117 patients in the propofol group and 122 patients in the remimazolam group before propensity score matching (PSM) (Figure 1).

The mean ± standard deviation induction doses were 14.50 ± 3.67 mg for remimazolam and 119.22 ± 23.74 mg for propofol before PSM. Baseline demographic and perioperative characteristics before and after PSM are summarized in Table 1. Before matching, several variables showed standardized mean differences (SMDs) greater than 0.2, indicating baseline imbalance between groups. After 1:1 propensity score matching, 99 matched pairs were generated. The covariates included in the propensity score model were adequately balanced, with absolute standardized mean differences < 0.1.

Postoperative outcome data are presented in Table 2. The primary outcome, the incidence of emergence agitation, was significantly lower in the remimazolam group than in the propofol group both before and after PSM. Before matching, emergence agitation occurred in 14 patients (11.5%) in the remimazolam group compared with 38 patients (32.5%) in the propofol group (p = 0.001). After matching, this difference remained significant, with emergence agitation observed in 13 patients (13.1%) in the remimazolam group and 36 patients (36.4%) in the propofol group (p = 0.001). After matching, conditional logistic regression analysis accounting for the matched-pair structure demonstrated that remimazolam-based induction was independently associated with a significantly reduced risk of emergence agitation (odds ratio 0.23, 95% confidence interval 0.09-0.56; p < 0.001).

**Figure 2:**
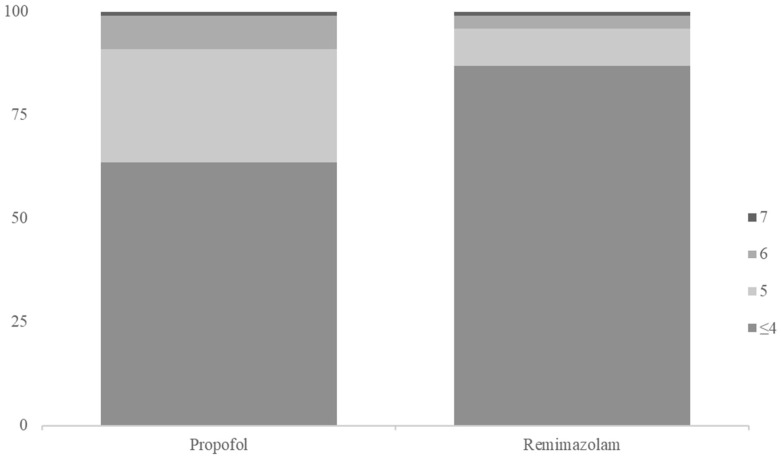
Distribution of Ricker Sedation-Agitation Scale (RSAS) scores after propensity score matching. Bars represent the proportion of patients in each RSAS category, with darker shading indicating greater agitation severity. The distribution of RSAS scores differed significantly between the remimazolam and propofol groups (p = 0.002). RSAS: Ricker Sedation-Agitation Scale.

The distribution of RSAS scores differed significantly between groups both before and after matching (Table 2). The post-matching distribution of RSAS scores is illustrated in Figure 2, demonstrating a shift toward a lower incidence and severity of emergence agitation in the remimazolam group.

Extubation time did not differ significantly between the propofol and remimazolam groups before or after matching. Postoperative pain scores measured by the NRS scores were also comparable between groups. The use of rescue analgesics in the post-anesthesia care unit tended to be less frequent in the remimazolam group; however, this difference did not reach statistical significance either before or after matching. The use of antiemetics in the post-anesthesia care unit was rare and did not differ between groups (Table 2).

Hemodynamic variables during emergence are summarized in **Supplementary Table 1**. After propensity score matching, systolic, diastolic, and mean blood pressure, as well as heart rate, at baseline, discontinuation of anesthetics, extubation, and 3 minutes after extubation were not significantly different between the propofol and remimazolam groups.

In univariate analysis, remimazolam-based induction, age, and sex were significant at p < 0.1 (Table 3). These variables were included in the multivariate logistic regression analysis. In the stepwise multivariate logistic regression analysis performed in the unmatched cohort, remimazolam-based induction was independently associated with a significantly lower risk of emergence agitation (adjusted OR 0.29, 95% CI 0.14-0.57; p < 0.001) (Table 4). Increasing age was independently associated with a reduced risk of emergence agitation (adjusted OR 0.97 per year, 95% CI 0.94-0.99; p = 0.001), whereas male sex was associated with a higher risk (adjusted OR 2.04, 95% CI 1.02-4.25; p = 0.049).

## Discussion

In this propensity score-matched retrospective study of adult patients undergoing elective transurethral endoscopic urologic surgery, remimazolam-based anesthetic induction was associated with a significantly lower incidence and severity of emergence agitation compared with propofol. This association was consistently observed before and after propensity score matching and remained significant in conditional logistic regression analysis accounting for the matched-pair structure. Importantly, the reduction in emergence agitation with remimazolam was not accompanied by prolongation of extubation time or deterioration in early postoperative recovery profiles, suggesting a favorable balance between agitation control and recovery efficiency.

Emergence agitation occurs during the transition from anesthetic-induced unconsciousness to full wakefulness, a period characterized by rapid changes in cortical activity and restoration of consciousness. Disruption in the regulation of cortical arousal during this transition has been implicated as a contributing mechanism to agitation during emergence from anesthesia 4, 15. In addition to central nervous system mechanisms, emergence agitation is frequently accompanied by peri-emergence hemodynamic changes, including sympathetic activation, hypertension, and tachycardia during early recovery 16, 17.

Several pharmacologic mechanisms may account for the lower incidence and severity of emergence agitation observed with remimazolam-based induction. Benzodiazepines are characterized by anxiolytic and amnestic properties that may persist into the early recovery phase, potentially attenuating disorientation and agitation during emergence from anesthesia 15. Consistent with this pharmacologic rationale, a randomized study in elderly patients undergoing hip replacement reported that remimazolam reduced the incidence of emergence agitation compared with propofol 18. Although benzodiazepine premedication has been suggested as a risk factor for emergence agitation in adults, the effect of benzodiazepines on emergence appears to be context-dependent, and perioperative administration of benzodiazepines reduced emergence agitation in both children and adults 5. In addition, benzodiazepine-based anesthetic techniques have been associated with smoother emergence profiles compared with non-benzodiazepine hypnotics, particularly when short-acting agents are used, without delaying recovery 1, 11.

Remimazolam, with its rapid esterase-mediated metabolism, may preserve these favorable emergence characteristics while minimizing the risk of prolonged sedation. Evidence from procedural sedation studies, including deep sedation during gastrointestinal endoscopy, suggests that remimazolam is associated with greater cardiovascular stability and fewer adverse events compared with propofol 8, 19, 20. Although smoother emergence may be accompanied by greater hemodynamic stability 13, blood pressure and heart rate during emergence did not differ significantly between the two groups after PSM. Therefore, our findings do not support a clear association between hemodynamic changes and the reduced incidence of emergence agitation.

Consistent with these pharmacologic considerations, the reduction in emergence agitation observed in the present study was achieved without prolongation of extubation time. In previous studies, the use of remimazolam for maintenance of anesthesia resulted in a delay in extubation time compared to maintenance of anesthesia with propofol or inhaled anesthetics 13, 21. Our finding supports the hypothesis that remimazolam, as an anesthetic induction agent, improves the quality of emergence rather than delaying awakening, thereby offering a clinically meaningful advantage in managing emergence agitation while preserving recovery efficiency.

In addition to the effect of the induction agent, younger age and male sex emerged as independent predictors of emergence agitation in the multivariate analysis. These findings are consistent with previous reports 1, 4, 15. Younger patients may be more susceptible to emergence agitation, possibly due to differences in the restoration of cortical activity and autonomic regulation during the transition from anesthesia to wakefulness. Similarly, male sex has been associated with heightened perioperative stress responses and behavioral disinhibition, which may contribute to agitation during early recovery. In addition, male sex is more susceptible to urinary catheter-related bladder discomfort than female sex, and urinary catheter-related bladder discomfort is an independent risk factor for emergence agitation 15, 22. The concordance between our findings and prior literature supports the clinical plausibility of the present results, although residual confounding cannot be excluded.

The clinical implications of our findings are particularly relevant in the context of urologic surgery. Patients undergoing urologic procedures frequently experience postoperative discomfort related to urinary catheterization, bladder distension, and surgical manipulation, all of which may predispose them to agitation during emergence from anesthesia 5. In addition, urologic surgical populations often include a higher proportion of male patients, a demographic that has consistently been identified as being at increased risk for emergence agitation 5,15. Occurrence of emergence agitation in this population may increase the risk of complications such as catheter dislodgement, surgical site bleeding, or unintentional removal of urinary drainage devices. Therefore, anesthetic strategies that reduce emergence agitation without delaying recovery or compromising hemodynamic stability are of clinical importance in urologic surgery. The favorable emergence profile observed with remimazolam-based induction in the present study suggests a potential role for this agent in optimizing perioperative management in this surgical population.

This study has several strengths. First, propensity score matching was applied to minimize baseline imbalances and reduce confounding related to anesthetic selection. Second, both the incidence and severity of emergence agitation were evaluated, providing a comprehensive assessment of emergence quality. Third, clinically relevant secondary outcomes, including extubation time and early postoperative recovery profiles, were examined, allowing assessment of potential trade-offs between agitation control and recovery efficiency. Finally, multiple complementary analytic approaches, including conditional logistic regression and multivariable modeling, were used to confirm the consistency and robustness of the findings.

However, several limitations should be acknowledged. First, the retrospective design limits the ability to establish causal relationships, and potential for selection bias and unmeasured confounding, despite the use of propensity score matching and multivariable adjustment exists. Second, emergence agitation was assessed based on routine clinical documentation, which may be subject to inter-observer variability. Third, this was a single-center study, and anesthetic practices, including induction protocols and postoperative management, may limit the generalizability of the findings to other institutions.

In conclusion, remimazolam-based anesthetic induction was associated with a significantly lower incidence and severity of emergence agitation compared with propofol in adult patients undergoing elective transurethral endoscopic urologic surgery. This benefit persisted after propensity score matching and matched-pair-adjusted analyses and was achieved without prolongation of extubation time or deterioration in early recovery profiles. These findings suggest that remimazolam may represent a favorable alternative to propofol for anesthetic induction in patients at risk for emergence agitation. Prospective randomized studies are warranted to confirm these findings and further elucidate the mechanisms underlying the observed benefits.

## Supplementary Material

Supplementary table.

## Figures and Tables

**Table 1:** Demographic data before and after propensity score matching.

	Before PSM				After PSM			
	Propofol group (n=117)	Remimazolam group (n=122)	p value	SMD	Propofol group (n=99)	Remimazolam group (n=99)	p value	SMD
Age (y)	60.74 (16.53)	57.56 (15.41)	0.125	0.199	59.46 (16.66)	58.66 (15.71)	0.726	0.050
Sex, male, n	76 (65.0)	61 (50.0)	0.027	0.306	60 (60.6)	58 (58.6)	0.885	0.041
Body mass index (kg/m^2^)	23.34 [21.97, 25.85]	24.70 [22.09, 26.90]	0.031	0.327	23.83 [22.09, 26.06]	24.14 [21.90, 26.42]	0.854	0.021
ASA physical status, n			0.556	0.141			0.906	0.063
I	5 (4.3)	5 (4.1)			4 (4.0)	4 (4.0)		
II	62 (53.0)	73 (59.8)			56 (56.6)	59 (59.6)		
III	50 (42.7)	44 (36.1)			39 (39.4)	36 (36.4)		
Duration of anesthesia (min)	75.00 [55.00, 110.00]	75.00 [56.25, 120.00]	0.707	0.015	75.00 [55.00, 120.00]	75.00 [55.00, 117.50]	0.843	0.097
Crystalloid administration (mL)	250.00 [150.00, 400.00]	225.00 [162.50, 350.00]	0.961	0.128	250.00 [175.00, 400.00]	200.00 [200.00, 350.00]	0.453	0.221
Estimated blood loss (mL)	15.00 [10.00, 20.00]	15.00 [10.00, 20.00]	0.625	0.076	15.00 [10.00, 20.00]	20.00 [10.00, 20.00]	0.616	0.181
Baseline systolic blood pressure, mmHg	147.90 (19.82)	150.47 (22.07)	0.345	0.123	148.00 (19.65)	151.85 (23.12)	0.208	0.179
Baseline heart rate, bpm	68.91 (12.61)	72.04 (12.19)	0.052	0.252	69.04 (12.57)	70.95 (11.75)	0.271	0.157
Postoperative PCA use, n	67 (57.3)	74 (60.7)	0.632	0.079	56 (56.6)	60 (60.6)	0.603	0.095

Values are expressed as mean (standard deviation), median [interquartile range], or number of patients (%). ASA, American Society of Anesthesiologists; PCA, patient-controlled analgesia; SMD, standardized mean difference.

**Table 2:** Comparison of postoperative outcomes before and after propensity score matching.

	**Before PSM**			**After PSM**		
	**Propofol group (n=117)**	**Remimazolam group (n=122)**	**p value**	**Propofol group (n=99)**	**Remimazolam group (n=99)**	**p value**
Emergence agitation, n	38 (32.5)	14 (11.5)	0.001	36 (36.4)	13 (13.1)	0.001
RSAS score			0.001			0.002
≤4	79 (67.5)	108 (88.5)		63 (63.6)	86 (86.9)	
5	28 (23.9)	9 (7.4)		27 (27.3)	9 (9.1)	
6	9 (7.7)	4 (3.3)		8 (8.1)	3 (3.0)	
7	1 (0.9)	1 (0.8)		1 (1.0)	1 (1.0)	
Extubation time, min	7.62 (3.07)	7.19 (2.96)	0.273	7.59 (3.22)	7.13 (2.88)	0.294
NRS pain score	3.26 (1.82)	3.20 (1.85)	0.789	3.32 (1.91)	3.14 (1.83)	0.487
Analgesics use in PACU, n	9 (7.8)	2 (1.6)	0.053	8 (8.1)	2 (2.0)	0.108
Antiemetics use in PACU, n	1 (0.9)	1 (0.8)	1.000	1 (1.0)	1 (1.0)	1.000
**Adjusted analysis after propensity score matching (conditional logistic regression)**						
			**Adjusted OR (95% CI)**		**p value**	
Emergence agitation associated with remimazolam-based induction (vs. propofol)			0.23 (0.09-0.56)		< 0.001	

Values are expressed as mean (standard deviation) or number of patients (%). PSM, propensity score matching; RSAS, Ricker Sedation-Agitation Scale; NRS, numeric rating scale; PACU, Post-anesthesia care unit.

**Table 3:** Univariate logistic regression analysis for emergence agitation.

	Unadjusted OR (95% CI)	p value
Remimazolam (vs. propofol)	0.28 (0.14-0.54)	<0.001
Age (per year)	0.97 (0.96-0.99)	0.01
Male sex	1.84 (0.97-3.64)	0.07
BMI (kg/m^2^)	1.03 (0.95-1.13)	0.45
ASA	0.86 (0.49-1.49)	0.59
Duration of anesthesia	1.00 (0.99-1.00)	0.59
Postoperative PCA use	0.80 (0.43-1.50)	0.48
Baseline systolic blood pressure	0.99 (0.97-1.00)	0.10
Baseline heart rate	0.98 (0.95-1.00)	0.11

BMI, body mass index; ASA, American Society of Anesthesiologists; PCA, patient-controlled analgesia; OR, odds ratio; CI, confidence interval

**Table 4:** Multivariate logistic regression for risk factors of emergence agitation before propensity score matching

	Adjusted OR (95% CI)	p value
Remimazolam (vs. propofol)	0.29 (0.14-0.57)	<0.001
Age (per year)	0.97 (0.94-0.99)	0.001
Male sex	2.04 (1.02-4.25)	0.049

OR, odds ratio; CI, confidence interval

## Data Availability

The data that support the findings of this study are available from the corresponding author upon reasonable request, subject to institutional and ethical restrictions.
