# Supplementary material for: Comparison of Emergence Agitation Between Remimazolam- and Propofol-Based Anesthetic Induction in Patients Undergoing Transurethral Endoscopic Urologic Surgery: A Propensity Score-Matched Retrospective Study
Source: Int J Med Sci. 2026 Jul 22;23(8):2739–46. doi: 10.7150/ijms.137664 (PMC13411052; doi:10.7150/ijms.137664)
Supplement: Supplementary file 1 — Supplementary table. [file ijmsv23p2739s1.pdf]

Supplementary Table 1. Hemodynamic variables before and during emergence after propensity score matching

Time point	Variable	Propofol group (n = 99)	Remimazolam group (n = 99)	P value	SMD
Baseline	SBP, mmHg	148.00 (19.65)	151.85 (23.12)	0.208	0.179
	DBP, mmHg	84.72 (10.09)	83.91 (10.29)	0.581	0.080
	MBP, mmHg	103.55 (10.51)	103.34 (10.82)	0.892	0.020
	HR, bpm	69.04 (12.57)	70.95 (11.75)	0.271	0.157
At discontinuation of anesthetics	SBP, mmHg	115.73 (17.82)	117.99 (17.82)	0.381	0.127
	DBP, mmHg	72.57 (12.53)	75.44 (16.53)	0.178	0.195
	MBP, mmHg	85.85 (12.80)	88.67 (14.74)	0.160	0.204
	HR, bpm	62.95 (13.20)	65.67 (18.00)	0.234	0.172
At extubation	SBP, mmHg	144.49 (24.93)	147.69 (25.58)	0.381	0.127
	DBP, mmHg	92.05 (19.41)	94.56 (17.06)	0.342	0.137
	MBP, mmHg	107.54 (19.19)	110.26 (16.68)	0.296	0.151
	HR, bpm	81.72 (18.08)	80.33 (15.46)	0.569	0.082
3 minutes after extubation	SBP, mmHg	144.09 (17.79)	149.16 (20.00)	0.065	0.267
	DBP, mmHg	88.23 (13.47)	91.41 (16.99)	0.153	0.207
	MBP, mmHg	104.95 (12.02)	108.23 (15.28)	0.100	0.239
	HR, bpm	78.03 (15.89)	79.28 (14.57)	0.571	0.082

Values are expressed as mean (standard deviation). PSM, propensity score matching; SBP, systolic blood pressure; DBP, diastolic blood pressure; MBP, mean blood pressure; HR, heart rate; SMD, standardized mean difference.
